# The effect of low-intensity resistance training after heat stress on muscle size and strength of triceps brachii: a randomized controlled trial

**DOI:** 10.1186/s12891-019-2991-4

**Published:** 2019-12-12

**Authors:** Masatoshi Nakamura, Tomoichi Yoshida, Ryosuke Kiyono, Shigeru Sato, Nobushige Takahashi

**Affiliations:** 1 Department of Physical Therapy, Niigata University of Health and Welfare, 1398 Shimami-cho, Kita-ku, Niigata City, Niigata 950-3198 Japan; 2 Institute for Human Movement and Medical Sciences, Niigata University of Health and Welfare, 1398 Shimami-cho, Kita-ku, Niigata City, Niigata 950-3198 Japan

**Keywords:** Low-intensity training, Ultrasound, Muscle thickness, Hot pack, One repetition maximum

## Abstract

**Background:**

The purpose of this study was to clarify whether there is a synergistic effect on muscular strength and hypertrophy when low-intensity resistance training is performed after heat stress.

**Methods:**

Thirty healthy young male volunteers were randomly allocated to either the low-intensity resistance training with heat stress group or the control group. The control group performed low-intensity resistance training alone. In the low-intensity resistance training with heat stress group, a hot pack was applied to cover the muscle belly of the triceps brachii for 20 min before the training. The duration of the intervention was 6 weeks. In both groups, the training resistance was 30% of the one repetition maximum, applied in three sets with eight repetitions each and 60-s intervals. The one repetition maximum of elbow extension and muscle thickness of triceps brachii were measured before and after 6 weeks of low intensity resistance training.

**Results:**

There was no significant change in the one-repetition maximum and muscle thickness in the control group, whereas there was a significant increase in the muscle strength and thickness in the low-intensity resistance training with heat stress group.

**Conclusion:**

The combination of heat stress and low-intensity resistance training was an effective method for increasing muscle strength and volume.

**Trial registration:**

University Hospital Medical Information Network Clinical Trials Registry (UMIN000036167; March 11, 2019).

## Background

In clinical settings, resistance training has been prescribed to prevent muscle atrophy and increase muscle mass. In general, high-intensity resistance training with at least 60–80% of one-repetition maximum (1RM) is recommended to increase muscle mass [1, 2]. However, studies have pointed out that high-intensity resistance training might be associated with a risk of orthopedic injury and that it further markedly increases systolic blood pressure [3–5].

Recently, studies have reported that low-intensity resistance training with blood flow restriction training or slow movements and tonic force generation induced a significant increase in muscle mass [6, 7]. In addition, it has to be considered that not only the training intensity but also the total work (training intensity × repetitions) performed is important to increase muscle mass. No significant difference has been found in the increase in muscle mass between low-intensity, high-repetition resistance training, and high-intensity resistance training when the total work was the same [8–10]. However, because it is necessary to increase the number of repetitions or contraction time in low-intensity resistance training to achieve the same total work as that in high-intensity resistance training [10], low-intensity, high-repetition resistance training is not a suitable approach in clinical settings because of the lack of required supervision and time constraint. Therefore, it is necessary to develop a more effective low-intensity resistance training method for this scenario.

In vitro studies have shown that heat stress can induce muscle hypertrophy [11, 12]. In animals, the effect of heat stress is independent of age [13]. An in vivo study demonstrated that heat stress causes an increase in both muscle mass and strength in young males [14]. Goto et al. investigated the combined effect of low-intensity resistance training with < 50% 1RM and heat stress in young males, and they could increase muscle mass and strength [15]. In the study by Yoon et al. on elderly women, they reported similar increases in muscle mass and strength with a combination of low-intensity resistance training (40% 1RM) and heat stress as obtained with moderate-intensity resistance training (60% 1RM) alone [16]. In contrast, Stadnyk et al. investigated the effect of heat stress during and after resistance training with 70% 1RM intensity in young subjects, and they reported that there were no effects on muscle mass and strength [17].

The lack of consensus on the effect of resistance training combined with heat stress in these studies may be related to differences in timing. When heat stress was applied during and after resistance training, as in the study by Stadnyk et al. (2017), no effect was observed. On the other hand, the application of heat stress before and during resistance training, as in the studies by Goto et al. (2007) and Yoon et al. (2017), achieved significant synergistic effects between resistance training and heat stress. Therefore, it might be important to apply heat stress before resistance training to induce an increase in muscle mass and strength.

The duration of the heat stress application was 1 [16] and 8 h [18] in previous studies, which is not realistic in a clinical setting. Moreover, the resistance training methods used in these studies were 50% 1RM × 30 repetitions × 3 sets [15] and 30% 1RM × 25 repetitions × 3 sets [16]. Therefore, it is possible that the increase in muscle mass and strength was in fact caused not by low-intensity but by high-repetition resistance training.

Therefore, we aimed to investigate the effect of low-intensity resistance training performed after heat stress, which was applied in a manner that is realistic in clinical settings (e.g., a heat stress duration of 20 min with 8 repetitions/set), on muscle mass and strength. Thus, the purpose of this study was to clarify whether low-intensity resistance training performed after heat stress has a synergistic effect on muscle strength and hypertrophy.

## Methods

### Subjects

In total, 30 healthy male volunteers who were nonathletes participated in this study [mean ± standard deviation (SD): age, 20.9 ± 0.4 years; height, 170.2 ± 5.3 cm; and weight, 62.8 ± 4.2 kg]. All subjects participated in sports at a recreational level and had not been involved in any regular resistance or flexibility training. In addition, the subjects were instructed to not start a new resistance or flexibility training during the training intervention period. Subjects with a history of neuromuscular disease or a musculoskeletal injury of the upper extremities were excluded. Written informed consent was obtained from all subjects. The study was approved by the Ethics Committee of the Niigata University of Health and Welfare, Niigata, Japan (17678), and it followed the CONSORT recommendations. The study was registered with the University Hospital Medical Information Network Clinical Trials Registry (UMIN000036167; March 11, 2019).

### Study protocol

**Fig. 1:**
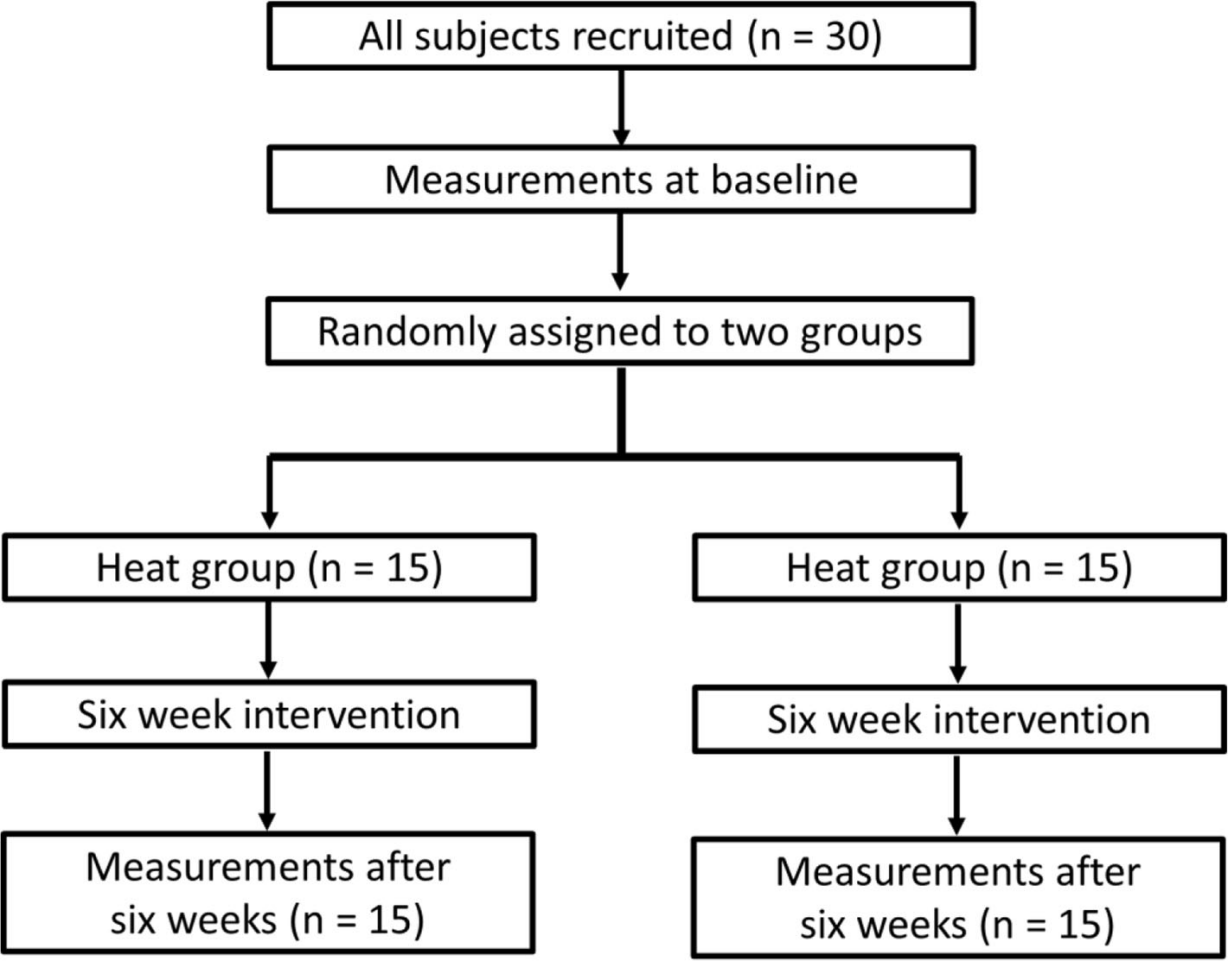
Flow chart of the study protocol

This study followed a randomized, controlled design (Fig 1). After baseline measurements, the subjects were randomly allocated to the combined low-intensity resistance training with heat stress group (heat group; *N* = 15) or control group (N = 15) using a computerized random number function in Microsoft Excel (Microsoft Corp., Washington, WA, USA). In the heat group, low-intensity resistance training was performed after 20-min heat stress. In the control group, low-intensity resistance training was performed without heat stress. For each subject, 1RM for elbow extension was measured 1 week before the training session, and the resistance training load was set to 30% 1RM. The 1RM measurements were repeated every 2 weeks during the intervention, and the resistance training load was readjusted based on the actual 1RM value.

### Resistance training program

The triceps brachii of the dominant arm was investigated in this study because previous studies have reported that the triceps brachii shows high responsiveness to resistance training aimed at an increase in muscle volume [19]. The resistance training comprised lying triceps extension with a dumbbell. The subjects laid in the supine position with the shoulder and elbow both in 90° flexion, and they were instructed to extend the elbow concentrically for 2 s, then eccentrically for 2 s, and finally isometrically for 1 s at an angle of 90°. In both groups, the training load was 30% 1RM and the resistance training comprised 3 sets with 8 repetitions and 60-s intervals. The resistance training was performed 3 days per week for 6 weeks (18 sessions).

### Muscle strength measurement

Each subject was instructed to perform a warm-up of 5 repetitions with a 3.5-kg dumbbell and 2 repetitions with a 5.0-kg dumbbell [20, 21]. After the warm-up, 1RM measurements were performed and the initial load was selected by each subject. The load was increased until the subject could not lift the weight anymore through a range of motion (elbow flexion from 90° to full extension) with the proper form. To avoid the effect of fatigue on 1RM, the rest period between two measurements was > 90 s. The 1RM measurements were performed at preintervention week 1 and postintervention weeks 2, 4, and 6.

### Measurements of muscle thickness

The thickness of the triceps brachii muscle was measured using B-mode ultrasonography (Aplio 500; Toshiba Medical Systems, Tochigi, Japan) with a 5–14-MHz linear probe. The measurement point was halfway on a line from the acromial process of the scapula to the lateral epicondyle of the humerus [20, 21]. The subjects were instructed to lie in the prone position on a desk with their arms placed at their sides and the wrist pronated. Measurements were taken from the inner edge of the fascia to the humerus. Muscle thickness was measured before and after resistance training intervention. Measurements were performed > 48 h after the last resistance training session to avoid errors due to acute edema. All measurements were performed by the same experienced investigator.

### Heat stress application

A hot pack was placed on the dominant upper arm for the application of heat stress. The subjects lied in the prone position, and the hot pack was applied to cover the muscle belly of the triceps brachii. Before application, the hot pack was heated to 75 °C in an hydrocollator and wrapped in a towel [18].

In a pilot study, we measured muscle temperature in 13 healthy males (mean ± SD: age, 21.2 ± 0.8 years; height, 171.7 ± 5.4 cm; and weight, 62.8 ± 4.3 kg) with a surface-type deep body thermometer (Core temp CTM-210; Telmo, Tokyo, Japan). The results showed that muscle temperature increased from 34.2 °C ± 1.0 °C (mean ± SD) before intervention to 36.9 °C ± 0.5 °C (mean ± SD) after low-intensity resistance training with heat stress using the same protocol as used in the heat group.

### Statistical analysis

SPSS (version 24.0; IBM Corp., Armonk, NY, USA) was used for statistical analysis. Potential differences between the heat and control groups for 1RM and muscle thickness before measurements were assessed with an unpaired *t*-test. For all variables, a split-plot analysis of variance (ANOVA) using two factors [group (heat vs. control group) and test time (before vs. after measurements)] was used to analyze the interaction and main effect. When a significant interaction was observed, the Bonferroni multiple comparison test was used to determine the differences in 1RM among the pre intervention measurements as well as postintervention weeks 2, 4, and 6 measurements. Paired *t*-test was used to determine the differences in muscle thickness between the before and after measurements in the heat and control groups. The differences were considered significant at an alpha level of 0.05. Descriptive data are shown as mean ± SD.

## Results

The changes in 1RM in both groups are presented in Table 1. No significant differences were found between these group in terms of 1RM before intervention using unpaired *t*-test (*p* = 0.873). In addition, split-plot ANOVA indicated a significant interaction effect (F = 5.012, *p* = 0.003, η_p_^2^ = 0.152). Regarding the heat group, post hoc tests revealed that 1RM at post intervention weeks 2, 4, and 6 was significantly higher than that before intervention. In addition, 1RM at post intervention week 6 was significantly higher than that at post intervention weeks 2 and 4. There were no significant differences in the control group in this regard.

**Table 1:** Muscle strength before, during, and after resistance training intervention

kg	PRE	2 weeks	4 weeks	6 weeks
Control group	11.6 ± 2.0	11.8 ± 1.9	12.2 ± 1.9	12.4 ± 2.1
Heat group	11.4 ± 2.4	12.4 ± 2.1^a^	12.9 ± 2.0^a^	13.8 ± 2.3^a, b, c^

Heat group: heat stress before training; PRE: before resistance training intervention

a: significant difference compared to PRE measurement

b: significant difference compared to measurement after 2 weeks

c: significant difference compared to measurement after 4 weeks

**Fig. 2:**
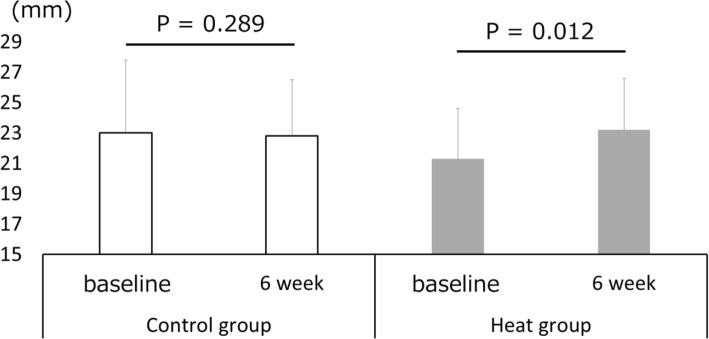
Muscle thickness before and after 6 weeks of resistance training. Control group: Resistance training only; Heat group: heat stress applied before resistance training; PRE: before resistance training intervention

The changes in the thickness of the triceps brachii in both groups are presented in Fig. 2. No significant differences were found between these group in terms of pre intervention measurements using unpaired *t*-test (*p* = 0. 299). In addition, the split-plot ANOVA indicated a significant interaction effect (F = 7.5, *p* = 0.011, η_p_^2^ = 0.211). The post hoc test revealed that muscle thickness after the intervention was significantly greater than that before the intervention in the heat group (*p* = 0.012), whereas there was no significant difference in muscle thickness before and after intervention in the control group (*p* = 0.289).

## Discussion

In this study, we investigated the effect of low-intensity resistance training combined with heat stress on the triceps brachii in healthy subjects. The main finding of this study was a significant increase in muscle strength and thickness after low-intensity resistance training (30% 1RM × 8 repetitions × 3 sets) preceded by 20-min heat stress, but no significant difference in terms of these parameters was observed after low-intensity resistance training alone.

Previous studies [14–16] that have investigated the effect of low-intensity resistance training with heat stress and reported increases in muscle strength and mass employed either long-duration heat application or high-repetition training. To the best of our knowledge, this is the first study to investigate the effect of low-intensity resistance training with heat stress on muscle strength and thickness wherein a mode of heat application and repetition that is practical in a clinical setting were used.

Goto et al. reported that heat application of 38 °C for > 45 min caused muscle hypertrophy [22], which might be due to the expression of heat-shock proteins caused by heat stress [23, 24]. It has been reported that heat stress supports muscle hypertrophy induced by mechanical stress [24, 25]. This suggests that in the heat group in our study, the effect of low-intensity resistance training was enhanced by the molecular chaperone function of heat-shock proteins after heat stress. This is supported by the finding that there was no significant change in muscle strength and thickness in the control group. However, a previous in vitro study reported that heat-shock protein 72 did not induce muscle hypertrophy at a muscle temperature of < 38 °C [22]. In our pilot study, we showed that muscle temperature increased from 34.2 °C ± 1.0 °C (mean ± SD) before intervention to 36.9 °C ± 0.5 °C (mean ± SD) after low-intensity resistance training with heat stress (no subject had muscle temperature of > 38 °C). Therefore, it is possible that there was no expression of heat-shock proteins after low-intensity resistance training combined with heat stress. Because we did not measure heat-shock proteins in this study, future studies are needed to measure these proteins to clarify the exact mechanism underlying the increase in muscle strength and thickness after low-intensity resistance training preceded by heat stress.

Remarkably, Goto et al. reported that heat stress alone increased both muscle strength and volume [14]. They applied heat stress for 6 h, and muscle temperature increased from 34.9 °C ± 0.5 °C (mean ± SD) to 38.3 °C ± 0.1 °C (mean ± SD) [14]. This muscle temperature of > 38.0 °C explains why heat stress alone could induce the described changes. On the other hand, in our study, heat stress was applied for only 20 min, which resulted in a muscle temperature of 36.9 °C ± 0.5 °C (mean ± SD). Therefore, we ascribe the observed positive effect on muscles to the combination of heat stress and low-intensity resistance training and not only to heat stress.

Generally, 60–80% 1RM training intensity is recommended to increase muscle strength and mass [1, 2]. However, previous studies have pointed out that high-intensity resistance training might be associated with a risk of orthopedic injury and marked increases in systolic blood pressure [3–5]. Therefore, low-intensity resistance training is preferable for clinical populations and the elderly. Our study suggests that low-intensity resistance training with heat stress is an effective training method for increasing muscle strength and mass in these target groups.

There are certain limitations to this study. First, we did not determine the nutritional status of the subjects. Thus, the true effectiveness of the training programs cannot be evaluated properly. Second, we investigated the effect of low-intensity resistance training with heat stress on the triceps brachii in healthy young males. A future study should investigate the effect of low-intensity resistance training after heat stress on lower limb muscles in clinical populations and the elderly.

## Conclusion

In this study, we investigated the effect of 6-week heat stress followed by low-intensity resistance training (30% 1RM × 8 repetitions × 3 sets) on the triceps brachii in young healthy males, and we showed a significant increase in muscle strength and thickness that was not observed with low-intensity resistance training alone.

## Data Availability

Data are available by contacting the corresponding author.

## Abbreviations

ANOVA: Analysis of variance
RM: Repetition maximum
